# A vision transformer based CNN for underwater image enhancement ViTClarityNet

**DOI:** 10.1038/s41598-025-91212-8

**Published:** 2025-05-14

**Authors:** Mohamed E. Fathy, Samer A. Mohamed, Mohammed I. Awad, Hossam E. Abd El Munim

**Affiliations:** 1 Mechatronics Engineering Department, Faculty of Engineering, Ain Shams University, Cairo, 11535 Egypt; 2 Computer and Systems Engineering Department, Faculty of Engineering, Ain Shams University, Cairo, 11535 Egypt; 3 Department of Electronic and Electrical Engineering, Faculty of Engineering and Design, University of Bath, Bath, BA2 7AY UK

**Keywords:** Convolutional neural networks, Generative adversarial, Underwater image enhancement, Vision transformer, Synthetic dataset, Engineering, Computer science, Software

## Abstract

Underwater computer vision faces significant challenges from light scattering, absorption, and poor illumination, which severely impact underwater vision tasks. To address these issues, ViT-Clarity, an underwater image enhancement module, is introduced, which integrates vision transformers with a convolutional neural network for superior performance. For comparison, ClarityNet, a transformer-free variant of the architecture, is presented to highlight the transformer’s impact. Given the limited availability of paired underwater image datasets (clear and degraded), BlueStyleGAN is proposed as a generative model to create synthetic underwater images from clear in-air images by simulating realistic attenuation effects. BlueStyleGAN is evaluated against existing state-of-the-art synthetic dataset generators in terms of training stability and realism. Vit-ClarityNet is rigorously tested on five datasets representing diverse underwater conditions and compared with recent state-of-the-art methods as well as ClarityNet. Evaluations include qualitative and quantitative metrics such as UCIQM, UCIQE, and the deep learning-based URanker. Additionally, the impact of enhanced images on object detection and SIFT feature matching is assessed, demonstrating the practical benefits of image enhancement for underwater computer vision tasks.

## Introduction

Unmanned underwater vehicles play a significant role in critical activities such as pipeline maintenance, underwater mining, fisheries management, and military surveillance^1,2^. Additionally, explorer-class unmanned vehicles are frequently employed to investigate geological formations, underwater archaeological sites, and marine ecosystems. Such vehicles require self- localization, visual perception and environment mapping to provide accurate feedback for trajectory control systems^3^. Visual perception tasks such as feature extraction, 3D image reconstruction, and autonomous navigation are suitable for cost-effective surface vehicle navigation^4–6^. However, dealing with turbulent underwater imagery often demands costly and power-intensive acoustic sensors such as the divergent-beam underwater Lidar imaging (UWLI) system^7^. This is attributed to the reduction of light intensity and light scattering, resulting in dimmer illumination and blurred images^8^. This reliance on complex sensors limits advancements in underwater navigation technology to some extent, contributing to the continued lack of exploration in most of the Earth’s water bodies^9^. Water’s greater density than air causes the gradual absorption of different wavelengths of light. Red light, with the longest wavelength, is absorbed first (within 10–15 feet). followed by orange (within 20–25 feet) and yellow (within 35–45 feet). This alteration of the original colors of the scene poses a challenge for object detection algorithms. Particularly those relying on color-based detection methods^10^. Several methods address underwater light attenuation, like Bazeille et al.’s approach^11^, which devised a detection pipeline that considers the light alterations from the source to the camera. Additionally, light refraction at media interfaces distorts epipolar lines, which makes traditional row-matching disparity estimation impractical. ZhuangS. et al.^12^ tackled this problem by presenting a model utilizing light field, where an underwater image is represented using direction-information and position images. Underwater conditions, characterized by limited visibility and degraded image quality, pose challenges to obtaining reliable visual features. Accordingly, conventional visual SLAM algorithms face challenges, prompting the need for innovative feature extraction and tracking tailored to underwater environments. Underwater attenuation-induced tracking errors can lead to inaccurate pose estimation, affecting map quality^13^.

Training machine learning models using an in-air dataset fails to generalize to underwater environments due to substantial domain variance. Additionally, proper annotation of underwater datasets is challenged by low contrast and color distortion, leading to limited-size datasets. These factors compound the difficulty of manually annotating underwater datasets^14^. Furthermore, existing machine learning methods for underwater tasks suffer notable performance degradation when faced with domain shifts caused by variations in light conditions and water quality across different water bodies, such as lakes and oceans. The restricted diversity of domains within the training data exacerbates this issue, as machine learning models tend to overfit to specific environments, failing to generalize to diverse water bodies. To address this challenge, some methods, such as the one proposed by Chen et al.^15^, enforce similar hidden features for images sharing identical semantic content across various domains.

This research addresses the limited availability of real underwater datasets by proposing a generative adversarial net- work capable of creating synthetic underwater datasets. Inspired by prior works like^16,17^. The proposed approach, named BlueStyleGAN, uses a GAN-based method for generating synthetic data, transferring underwater visual style from specific scenes to annotated in-air images. Unlike WaterGAN’s simplistic architecture, the proposed approach employs a more complex model-based architecture to accurately capture underwater attenuation. Additionally, unlike Ye et al.’s method^17^, which utilizes depth map information during training—hindering GAN convergence—the proposed approach simplifies the process by only requiring pairs of underwater and above-water RGB images, eliminating the need for depth maps during training. This simplification reduces both training time and effort. Depth maps are integrated post-training to fine-tune underwater effects as necessary. Furthermore, our approach modifies the GAN architecture to solely adopt style transfer techniques proposed by Gatys et al. in^18^, contrasting with Ye et al.’s method^17^ that incorporates both style and content transfer. The generated synthetic dataset are used for training and evaluating machine learning techniques intended for underwater use. In this study, the generated dataset is utilized to train image enhancement modules. These modules employ encoder-based architectures, with one integrating a vision transformer (ViT)^19^ for feature extraction.

The paper is structured as follows: Related Works reviews recent literature; Methods details the methodology; Results presents the findings and their analysis in the Discussion; Conclusion summarizes the work.

## Related works

Image enhancement techniques can be categorized into different categories. The first category is conventional methods like gamma correction and histogram equalization^20^, which are notably constrained for such tasks. This is because degradation in underwater images is both additive and multiplicative. Moreover, these methods ignore the importance of range-based degradation. As a result, pixels from the same object may lose photometric consistency, altering the object’s visual features and posing a challenge for computer vision algorithms that rely on feature matching, such as object detection and stereo matching.

The second category consists of model-based methods that employ physical models accounting for range-dependent attenuation, like the approach by Wang and Wu^21^. They relied on the Jaffe-McGlamery model, considering RGB-D images as a comprehensive representation of both photometric and geometric aspects, enabling better characterization of light attenuation behavior. However, acquiring model parameters requires prior knowledge of the full-depth map and specific experiments at the survey site. This method struggles with generalization due to its simplicity and the need for repeated experiments with varying water conditions.

The last category is the data-driven approach, which addresses the generalization challenge and captures more complex mod- els by training neural networks end-to-end. This category offers the advantage of adaptability, allowing the models to adjust to variations in water characteristics. For instance, Chongyi Li et al.^22^ proposed WaterNet, which is a CNN trained on the proposed UIEB dataset. Ancuti et al.^10^ employed a multi-scale fusion strategy to enhance global contrast and edge sharpness in images derived from a white-balanced single input image. Peng et al.^23^ utilized image blurriness to estimate depth maps for enhancing visual images. Peng et al.^24^ proposed a method for underwater depth estimation based on image blurriness and light absorption, aimed at enhancing underwater images. However, obtaining a sufficiently large dataset of real underwater attenuated images along with corresponding ground-truth scenes after water removal is impractical. To address this challenge, several methods have been proposed. Skinner et al. introduced UWStereoNet^25^ to eliminate the need for annotated underwater datasets using unsupervised learning. Similarly, Yin et al.^26^ applied contrastive learning to address the absence of paired distorted and reference images by treating distorted underwater images as negative examples and clear images as positive examples. Additionally, Song et al. incorporated reinforcement learning into the model’s fine-tuning phase, where the reward function is based on three no-reference quality metrics: UCIQE^27^, NIQE^28^, and URanker^29^. However, lack in supervision can cause lower performance due to increased uncertainty and variability in learned patterns. This has led to the development of synthetic underwater dataset generators. For instance, Li et al.^30^ proposed UWCNN, an underwater image enhancement CNN model. It synthesizes underwater distortions to create a training dataset, and after that, it is trained to reconstruct clear underwater images while preserving structure and texture. Additionally,^16,17,31–33^ employed GAN-based networks to create underwater-distorted versions of clear images through style transfer. These generated datasets were then used for paired training.

Skinner et al. introduced WaterGAN^16^, a GAN-based network generating synthetic underwater images from RGB-D in-air and RGB underwater inputs. The resulting dataset is used to train a CNN for monocular depth map estimation and color restoration. Due to the simplifications made for GAN training stability, WaterGAN’s realism and customization of the generated underwater scenes are diminished. Cui et al.^31^ and Cameron Fabbri et al.^32^ applied CycleGAN^34^ to perform style transfer, generating synthetic underwater-distorted images from clear images. However, CycleGAN lacks tunable multiple-style transfer weights. Additionally, the absence of explicit annotations can lead to inconsistencies. Moreover, the exclusion of depth maps allowed the usage of CycleGAN as a data generator, but it also precluded the synthesis of depth-dependent attenuation. For Cui et al., this limitation affects the disparity estimation network’s learning capacity, as it cannot utilize pixel attenuation as an additional clue for predicting depth. Ye et al.^17^ used an adversarial training framework to create a synthetic underwater dataset from an RGB-D in-air dataset. They also incorporated Gatys et al.’s style transfer approach^18^, applying content loss to both generated and in-air images, along with style loss for generated and underwater images. This improved training convergence compared to WaterGAN. The resulting dataset was then used to jointly train depth estimation and color correction modules. Gonzalez-Sabbagh et al.^35^ directly employed adversarial training for image enhancement by introducing a dual-generator, single-discriminator architecture, where one generator enhances distorted images, and the other simulates the imaging process using transmission information, enabling multi-scale information integration.

## Methods

This study aims to mitigate underwater attenuation by training ViT-ClarityNet, an encoder-decoder convolutional neural network (CNN) that incorporates the vision transformer proposed by Alexey Dosovitskiy et al.^19^ for efficient feature extraction, which has proven to significantly enhance overall performance. To obtain the annotated training dataset, manually acquiring undistorted versions of actual underwater scenes is impractical, necessitating the use of synthetic data generation. This is achieved through a style transfer module called BlueStyleGAN. BlueStyleGAN is trained to generate an image ($i_{g}$) by combining an in-air image ($i_{a}$) and an underwater image ($i_{w}$) from the respective distributions $I_{a}$ and $I_{w}$. The resulting image ($i_{g}$) should preserve the content of the in-air image ($i_{a}$), such as objects and structures, while incorporating the visual style of the underwater image ($i_{w}$).

### Synthetic data generation

**Fig. 1:**
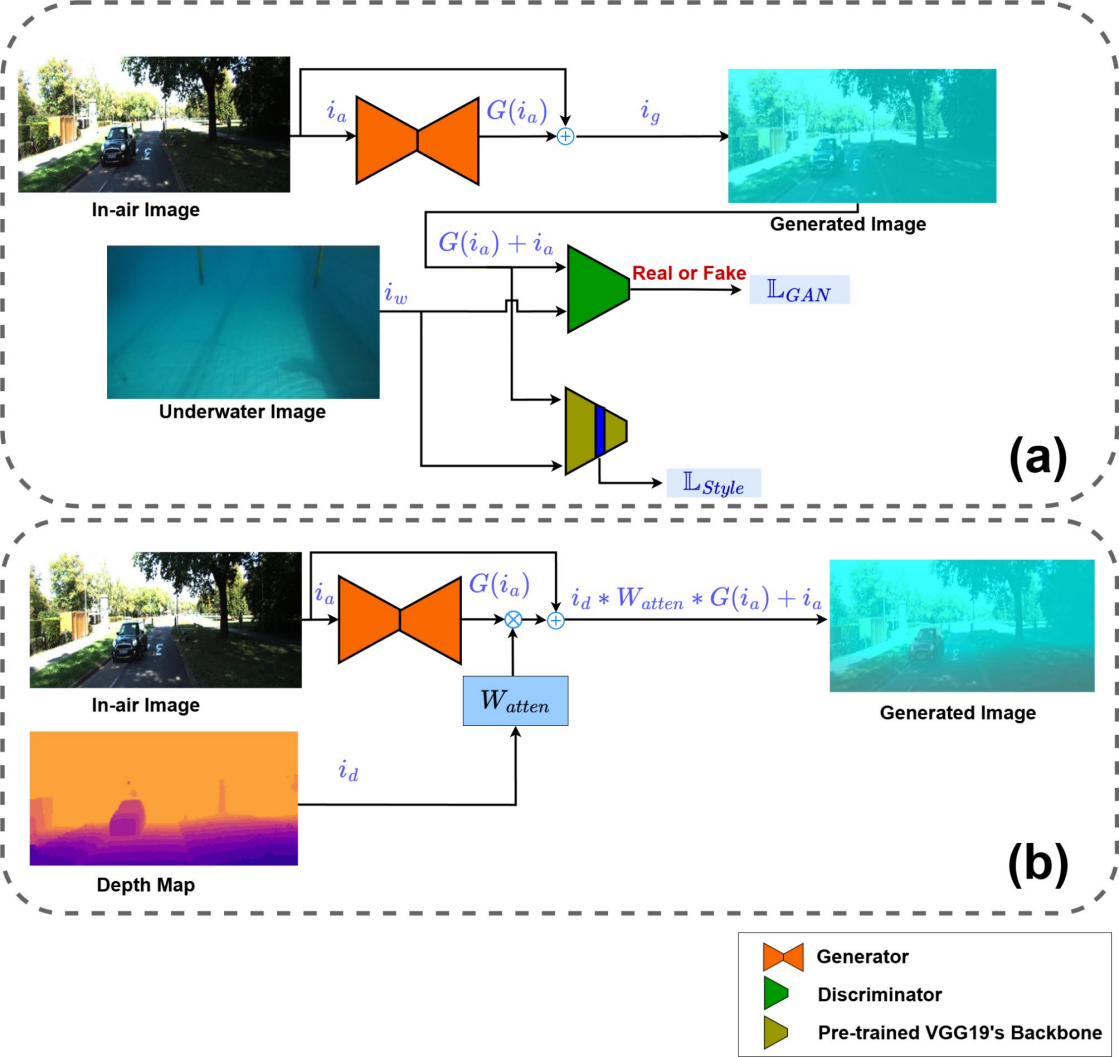
BlueStyleGAN architecture for (**a**) training stage and (**b**) inference stage.

BlueStyleGAN is a generative adversarial network (GAN). Figure 1a shows the training stage, where the generator *G* takes an in-air image $i_{a}$ from the distribution $I_{a}$ and is trained to obtain an underwater attenuation style $G\left( {i_{a} } \right)$. This style is added to the original image to create a synthetic underwater image $i_{g}$ = $G\left( {i_{a} } \right) + i_{a}$. The discriminator *D* is trained to classify the generated image $i_{g}$ as fake while classifying a real underwater image $i_{w}$ as real. The adversarial loss function ${\mathbf{L}}_{GAN}$ is defined as follows:

1 $$\mathbf{L}_{GAN}=\mathbb{E}_{i_{w}\sim I_{w}}[\log{D(i_w)}] +\ \mathbb{E}_{i_{a}\sim I_{a}}[1 - \log{D(G(i_a) + i_a)}]$$

To stabilize the GAN’s training and ensure homogeneity in underwater style across the image the style transfer method proposed by Gatys et al.^18^ is incorporated, adding an additional loss function ${\mathbf{L}}_{Style}$, which aligns the Gram matrix corresponding to the generated image $i_{g}$ with that of the underwater image $i_{w}$. Computing the Gram matrix requires feature extraction, which is performed using the backbone of a VGG19 network pre-trained on the ImageNet dataset^36^. Features are extracted from both $i_{g}$ and $i_{w}$, obtaining feature maps $f_{g}$ and $f_{w}$, respectively. Experiments reveal that the 4th and 5th layers of the VGG19 model capture the desired style features. The ${\mathbf{L}}_{Style}$ loss is defined as follows:

2 $$\mathbf{L}_{\text{Style}} = \sum_{l \in \{4,5\}} w^l \left\| \mathbb{G}^l(f_w) - \mathbb{G}^l(f_g) \right\|_{F}^2$$

The proposed approach is similar to that introduced by Ye et al.^17^, but with several key differences. The generator in the proposed approach is trained solely to produce the attenuation style $G\left( {i_{a} } \right)$, which is then added to the original images. This results in two main differences that simplify and stabilize the training process: first, it eliminates the need for content loss since the generator only produces the distortion style; second, it omits skip connections in the generator’s encoder-decoder as learning high-resolution content is unnecessary. Another notable difference from Ye et al.^17^ is the exclusion of in-air depth maps from the training procedure. Instead, the attenuation style is applied uniformly, disregarding depth dependency, which enhances training stability. This is justified by the unavailability of underwater depth maps, which hinders the discriminator’s ability to realize the correlation between attenuation and depth, so it won’t be able to judge if the generator is using the in-air depth map correctly. Accordingly, the generator won’t be able to use the depth map correctly to apply appropriate depth-dependent distortion. During the inference stage (as shown in Fig. 1b), depth maps are used to implement adjustable range-dependent attenuation using the parameter *W*_*atten*_. This enables adaptation to various attenuation levels by adjusting a tunable parameter without re-training the entire module. Furthermore, this approach enables acceptable style transfer even when in-air depth maps are unavailable.

BlueStyleGAN’s generator uses the same architecture as that of CycleGAN^34^. The discriminator consists of four convolu- tional layers with batch normalization, leaky ReLU activation, and average pooling. The output feature maps have 64, 128, 256, and 512 channels, respectively. The flattened output is processed by three fully connected layers with 1024, 512, and 64 neurons using leaky ReLU activations with final sigmoid activation for classification.

### Image enhancement

**Fig. 2:**
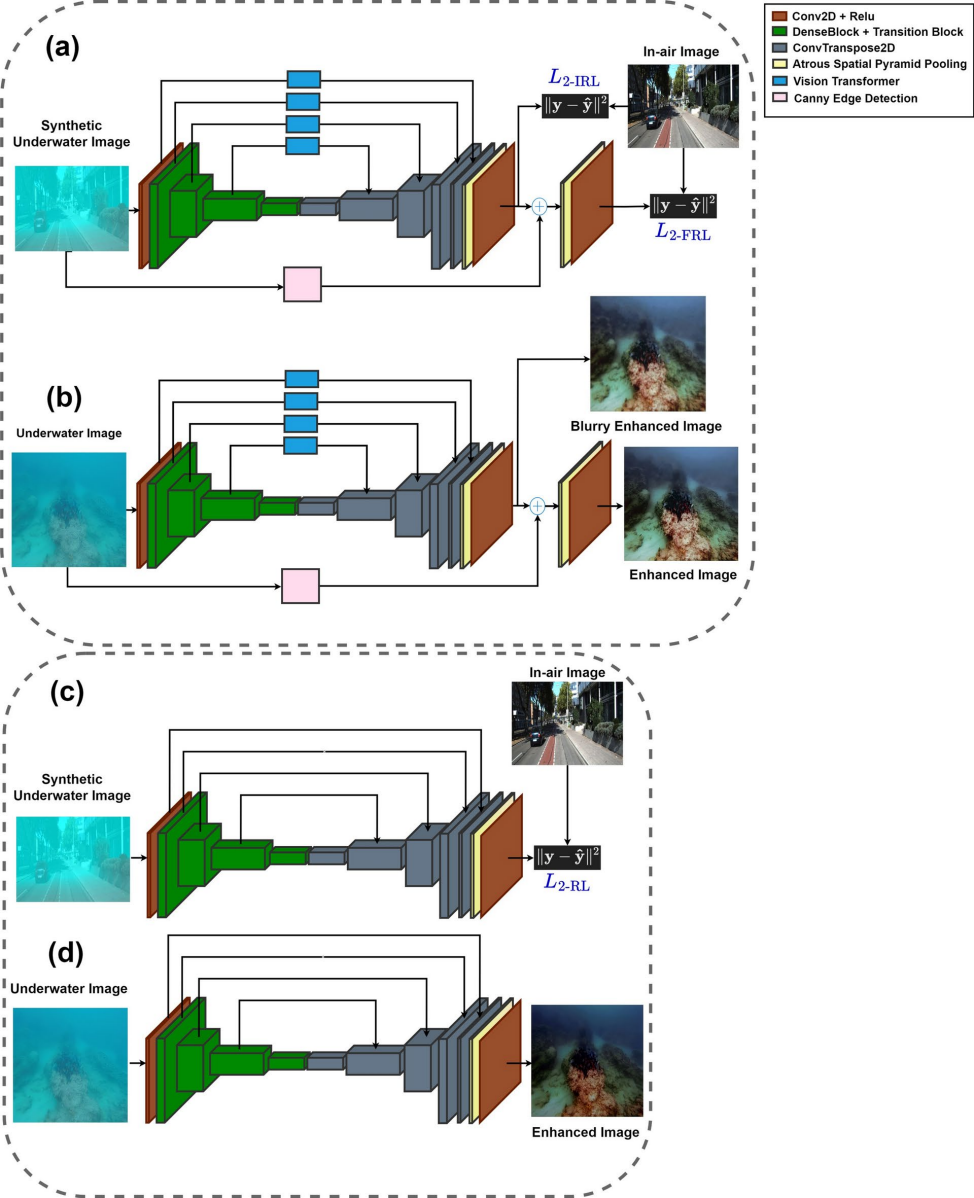
Architectural diagrams of the proposed Image Enhancement Modules. (**a**) and (**b**) show ViT-ClarityNet during training and inference stages, respectively. (**c**) and (**d**) show ClarityNet during training and inference stages, respectively.

The aim of the Image Enhancement Module (Fig. 2) is to counteract the effects of water attenuation, producing enhanced images with enhanced contrast and clearer edges. This module is trained using a dataset of attenuated images ${i}_{g}$ generated by BlueStyleGAN, alongside original in-air images ${i}_{a}$ provided as ground truth reference.

In this study, two encoder-decoder CNN architectures are introduced. The first architecture, ViT-ClarityNet (shown in the upper diagram of Fig. 2 as (a) for training and (b) for inference), employs vision transformers in the skip connections for more efficient feature extraction. Vision transformers have proven to be superior feature extractors, especially in our scenario where pixels within a scene experience variable attenuation due to differences in distance and photometric characteristics. Their large receptive field effectively captures comprehensive global contextual information to counteract and mitigate these effects. However, vision transformers may lose high-frequency local details while capturing extensive global contextual information, leading to blurred images. To address this issue, a differentiable Canny edge detection module is employed to generate an edge detection image. This edge detection image is added to the blurred image. The combined output is then refined through a convolutional layer, enhancing edge sharpness. In order to guarantee effective use of Canny Edge detection, it must be ensured that an appropriate initial RGB image is added to the edge detection image. For this reason, the initial output is subjected to the L2 loss function called Initial Reconstruction Loss *L*_2-IRL_. Subsequently, the Final Reconstruction Loss *L*_2-FRL_ is applied to the final output to refine the image with sharp edges. The total reconstruction loss *L*_2-RL_, which weights the two loss functions with *W*_*I*_ and *W*_*F*_ respectively, is shown in Eq. (3), The second architecture, ClarityNet (shown in the lower diagram of Fig. 2 as (c) for training and (d) for inference), employs a single L2 loss function for the final output.

3 $$L_{{{2} - {\text{RL}}}} = W_{I} \cdot L_{{{2} - {\text{IRL}}}} + W_{F} \cdot L_{{{2} - {\text{FRL}}}}$$

Both Vit-ClarityNet and ClarityNet adopt an encoder-decoder architecture. The encoder initiates with a convolutional layer producing a 48-channel feature map; after that, four dense block modules are employed, as proposed by Huang et al.^37^, where each block comprises three dense layers with a growth rate of 12. A transition block follows each dense block to reduce concatenated input channels, resulting in feature maps of channel sizes 96, 192, and 384, respectively. Each transition layer is supplemented with batch normalization and ReLU activation. The decoder includes three transposed convolutional layers that upsample feature maps while reducing channel sizes (384 to 192, 192 to 96, and 96 to 48). An Atrous Spatial Pyramid Pooling (ASPP) module by Chen et al.^38^ is applied after the third decoder layer to capture multi-scale contextual information. Finally, a convolutional layer converts 48 input channels to 3 for the final RGB image.

ViT-ClarityNet integrates four vision transformers, pretrained on CIFAR10, as additional blocks within the skip connections between the encoder and decoder layers. These transformers act as powerful feature extractors and are fine-tuned during the training of ViT-ClarityNet to adapt specifically to underwater images in a transfer learning framework. Each transformer is characterized by a hidden state dimension of 768, an MLP dimension of 3072, 12 attention heads, and 12 layers. The transformers process input feature maps in batches with sizes of 32×32, 16×16, 8×8, and 4×4.

### Training and inference details

#### Synthetic data generation

For synthetic dataset preparation, BlueStyleGAN was trained using in-air RGB-D images sourced from the KITTI dataset^39^, along with an underwater dataset consisting of 250 samples obtained from a test pool, as shown in Fig. 1a. The training process converged in just 15 epochs. The style loss, as shown in Eq. (2), was applied starting from the 2nd epoch, with $\omega^{l}$ set to 500 for both the 4th and 5th layers. A batch size of 8 and image dimensions of 640 × 480 were used. The Adam optimizer was employed with parameters $\beta$_1_ = 0*.*5 and $\beta$_2_ = 0*.*999, and a learning rate of 3 × 10^*−*4^.

During inference, as shown in Fig. 1b, depth maps were used to apply range-dependent attenuation. For numerical stability, the maximum depth map was set to 15 meters, and *W*_*atten*_ was adjusted based on the desired attenuation style.

#### Image enhancement

Using the synthetic underwater dataset generated by BlueStyleGAN, along with its corresponding in-air images as ground truth (see Fig. 2a and c), both ViT-ClarityNet and ClarityNet were trained under identical conditions. The training was held for 80 epochs with a batch size of 4 and image dimensions of 640 × 480. The Adam optimizer was used with parameters $\beta$_1_ = 0*.*5 and $\beta$_2_ = 0*.*999 with a learning rate of 3 × 10^*−*4^. For ViT-ClarityNet’s training, the loss function weights in Eq. (3) were set to *W*_*I*_ = 0*.*3 and *W*_*F*_ = 0*.*7.

## Results

### Synthetic data generation

**Fig. 3:**
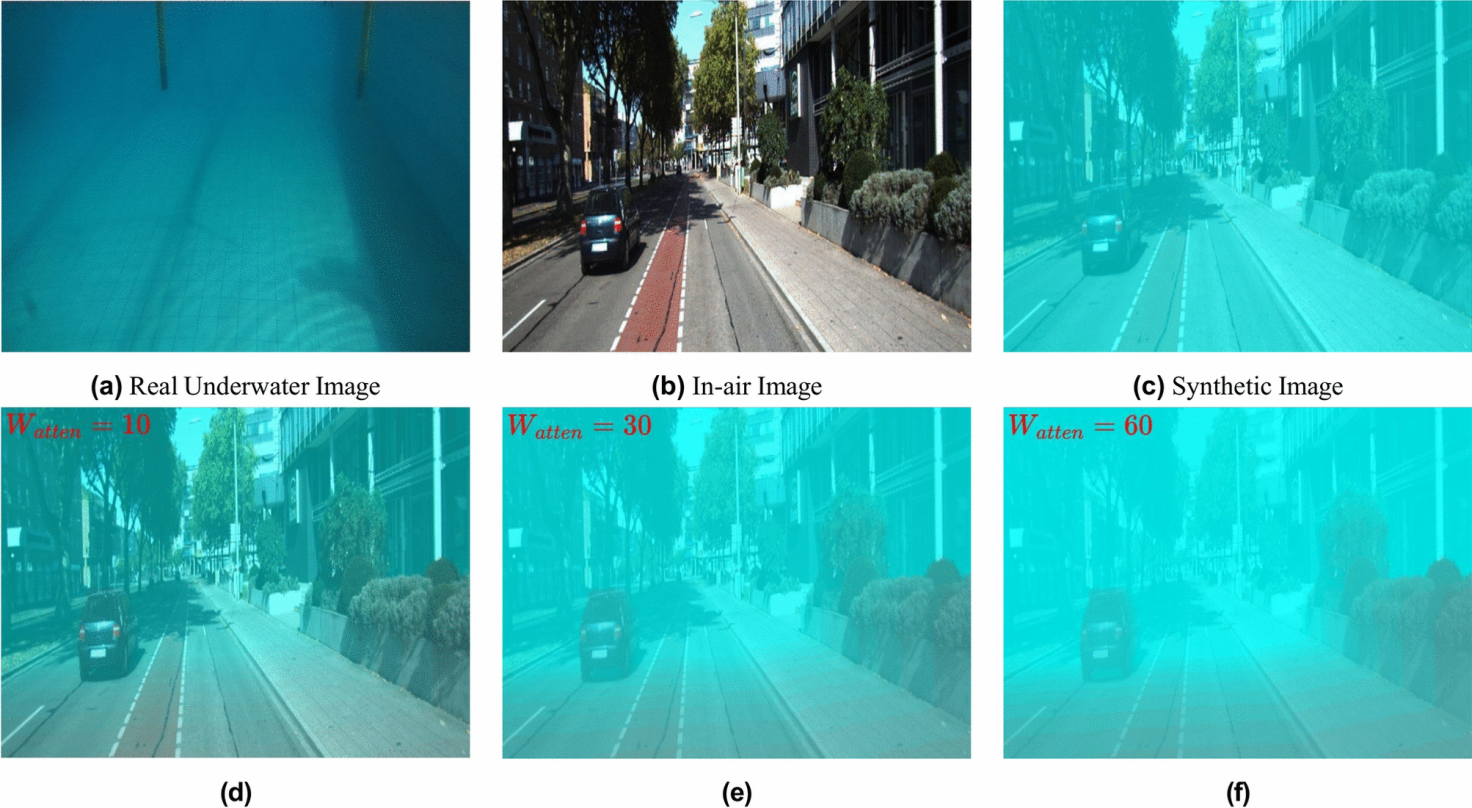
Top row: Results obtained during the training stage, showing the generation of a synthetic underwater image (**c**) by merging the style of a real underwater image (**a**) and the content of an in-air image (**b**). Bottom row: Results (**d**), (**e**), and (**f**) obtained by varying *W*_*atten*_ (10, 30, and 60, respectively) during the inference stage to achieve different attenuation levels.

The effectiveness of BlueStyleGAN is evaluated by showing its capability to achieve diverse attenuation styles using depth maps simply by adjusting the weight *W*_*atten*_ during the inference stage, unlike WaterGAN^16^ and Ye et al.^17^, which require retraining for such variations. As shown in Fig. 3, image (c) shows the result without using the depth map, while the bottom row images display results with *W*_*atten*_ set to 10, 30, and 60, respectively.

**Fig. 4:**
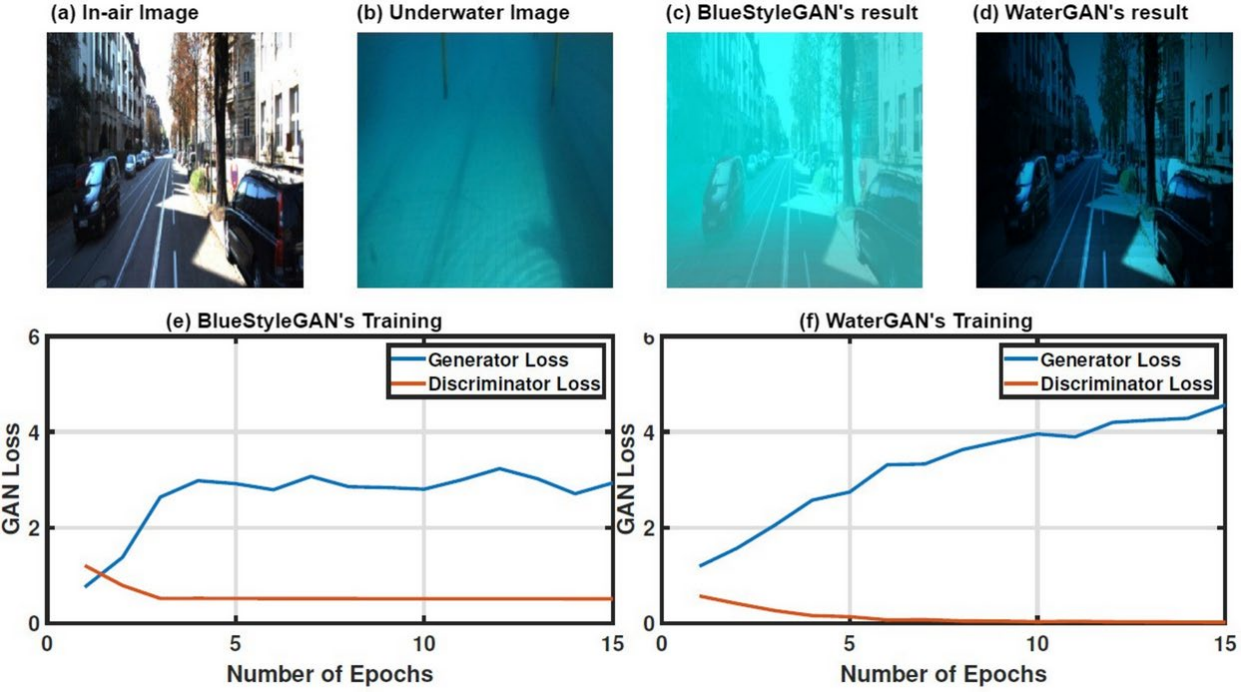
A qualitative comparison between BlueStyleGAN and WaterGAN^16^ regarding generated synthetic data and the convergence of training loss curves.

The stability of BlueStyleGAN’s training process and the quality of its synthetic images are also evaluated in comparison to WaterGAN^16^. Figure 4 indicates that BlueStyleGAN shows indications of convergence in both generator and discriminator losses, suggesting a more stable and effective training process than WaterGAN^16^. Moreover, when generating synthetic underwater images, BlueStyleGAN achieves a more realistic style transfer from the provided underwater environment.

**Fig. 5:**
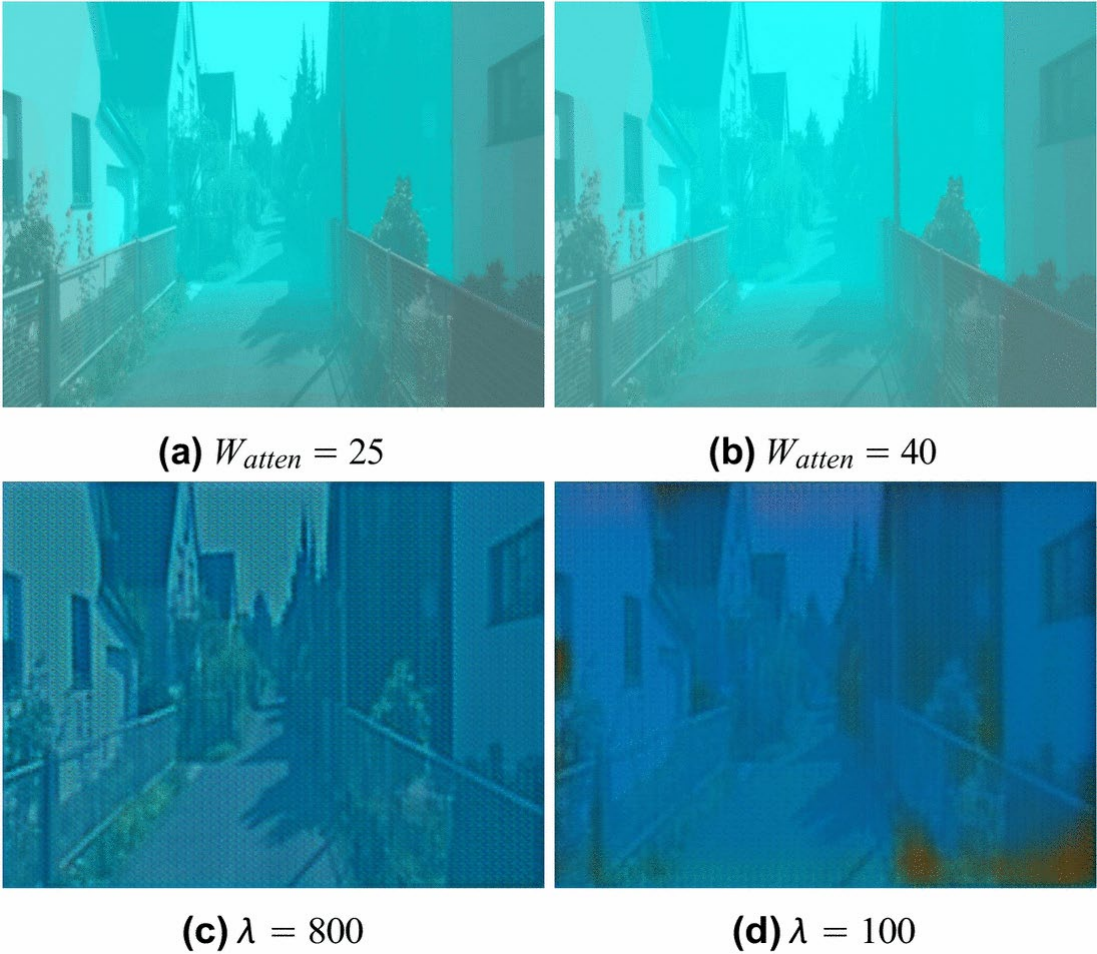
Comparison of style transfer performance between BlueStyleGAN (first row) and CycleGAN (second row). (**a**) and (**b**) show the style transfer results of BlueStyleGAN with different depth map attenuation weights *W*_*atten*_ = 25 and *W*_*atten*_ = 40, respectively, while (**c**) and (**d**) show the results of CycleGAN with varying cyclic consistency loss weights *λ* = 800 and *λ* = 100, respectively.

BlueStyleGAN employs the same generator architecture as CycleGAN^34^ but uses it in a different manner. To highlight the impact of this difference, Fig. 5 illustrates a comparison of the performance of BlueStyleGAN (top row) and CycleGAN^34^ (bottom row) as style transfer modules. For BlueStyleGAN, the style transfer effect is controlled by adjusting the depth map attenuation weight *W*_*atten*_, whereas in CycleGAN^34^, the effect depends on the cyclic consistency loss weight *λ*. At lower *λ* values (e.g., *λ* = 100), CycleGAN^34^ introduces noticeable color artifacts due to a reduced weight in preserving the structure of the original in-air domain. The generator in CycleGAN^34^ also struggles to produce high-quality outputs with fine details, highlighting architectural limitations. This limitation justifies BlueStyleGAN’s approach of using the same architecture differently by generating only attenuation hues without the need to create fine details. Furthermore, CycleGAN^34^ requires retraining for each new style transfer task and cannot incorporate distance-dependent effects using depth maps. These limitations highlight the superior efficiency and flexibility of BlueStyleGAN compared to CycleGAN^34^.

### Image enhancement

The chosen benchmark for evaluating the proposed Image Enhancement modules (ViT-ClarityNet and ClarityNet) aims to validate the motivation behind this research, which addresses the mitigation of limitations of underwater computer vision compared to in-air scenarios, which are due to poor textural and color features.

**Fig. 6:**
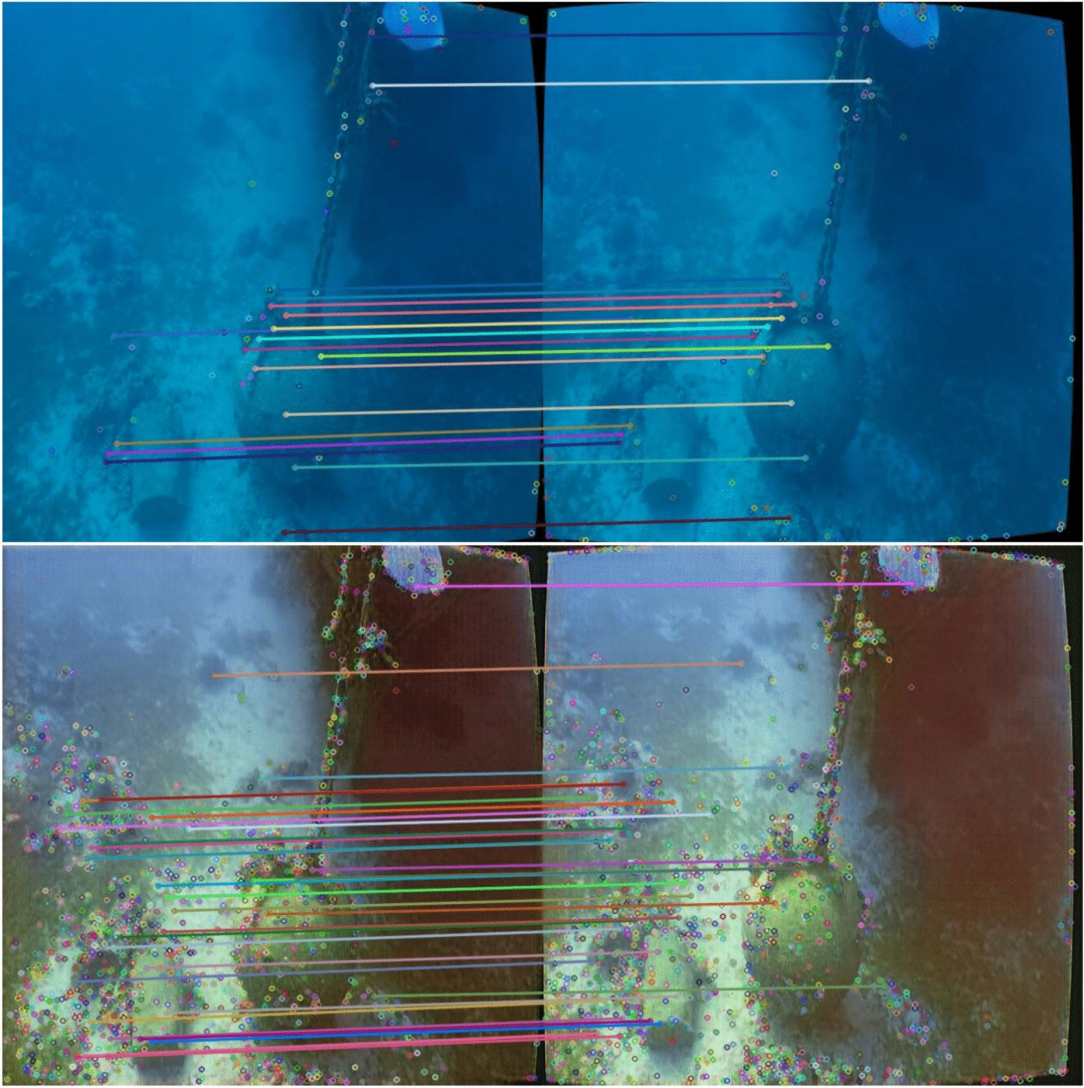
The Influence of Image Enhancement Using ViT-ClarityNet on Scale-Invariant Feature Transform (SIFT) Matching.

Firstly, the performance of ViT-Clarity feature sharpening is assessed using Scale Invariant Feature Transform (SIFT) matching, shown in Fig. 6 with a sample from the SQUID dataset^40^.

**Fig. 7:**
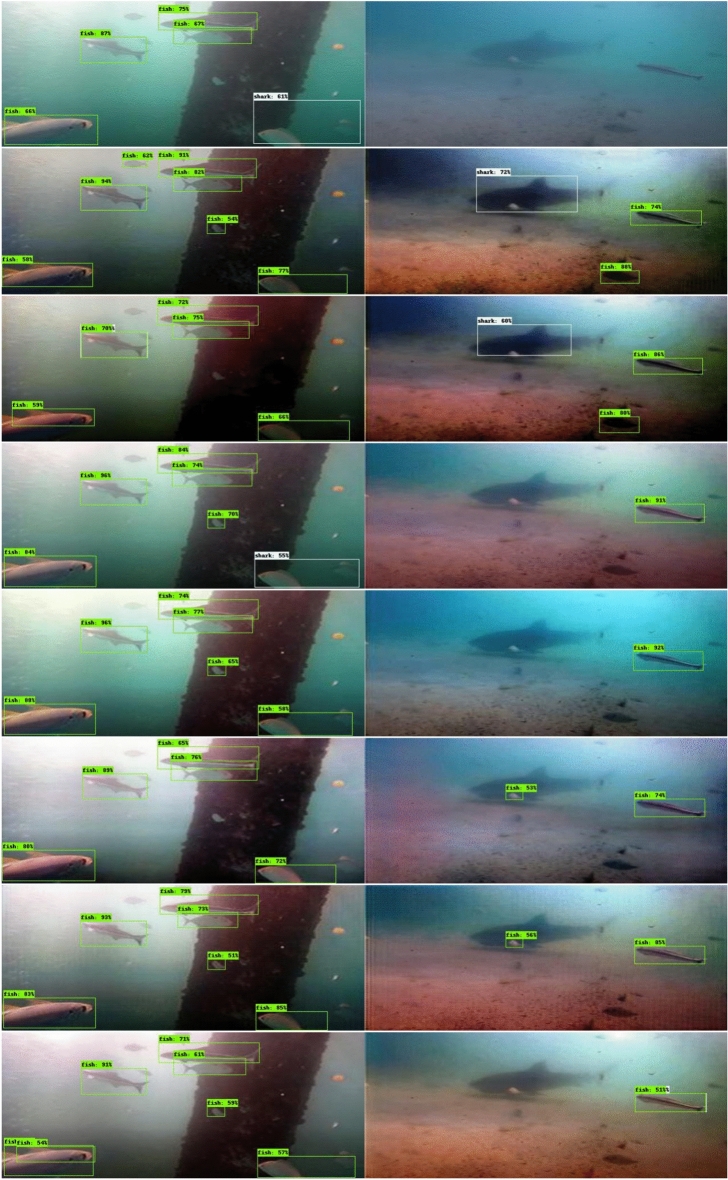
Performance comparison of object detection techniques using images from original underwater, ViT-ClarityNet, ClarityNet, WaterNet^22^, Deep WaveNet^43^, FUnIE-GAN^33^, UGAN^32^, and RAUNE-Net^44^. The fish detections are indicated with green boxes, while shark detections are indicated with white boxes.

Secondly, as shown in Fig. 7, the evaluation benchmark illustrates the performance of the EfficientDet model^41^, trained on the Aquarium dataset^42^, in detecting objects in foggy underwater scenes sourced online and in enhanced images.

**Fig. 8:**
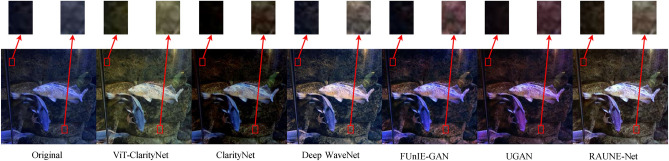
Visual and quantitative comparison (considering CM, SM, ConM, UIQM, URanker, and UCIQUE metrics) for brightness variance on a sample from the Aquarium dataset^42^. From left to right: raw underwater images, ViT-ClarityNet, ClarityNet, Deep WaveNet^43^, FUnIE-GAN^33^, UGAN^32^, and RAUNE-Net^44^.

**Fig. 9:**
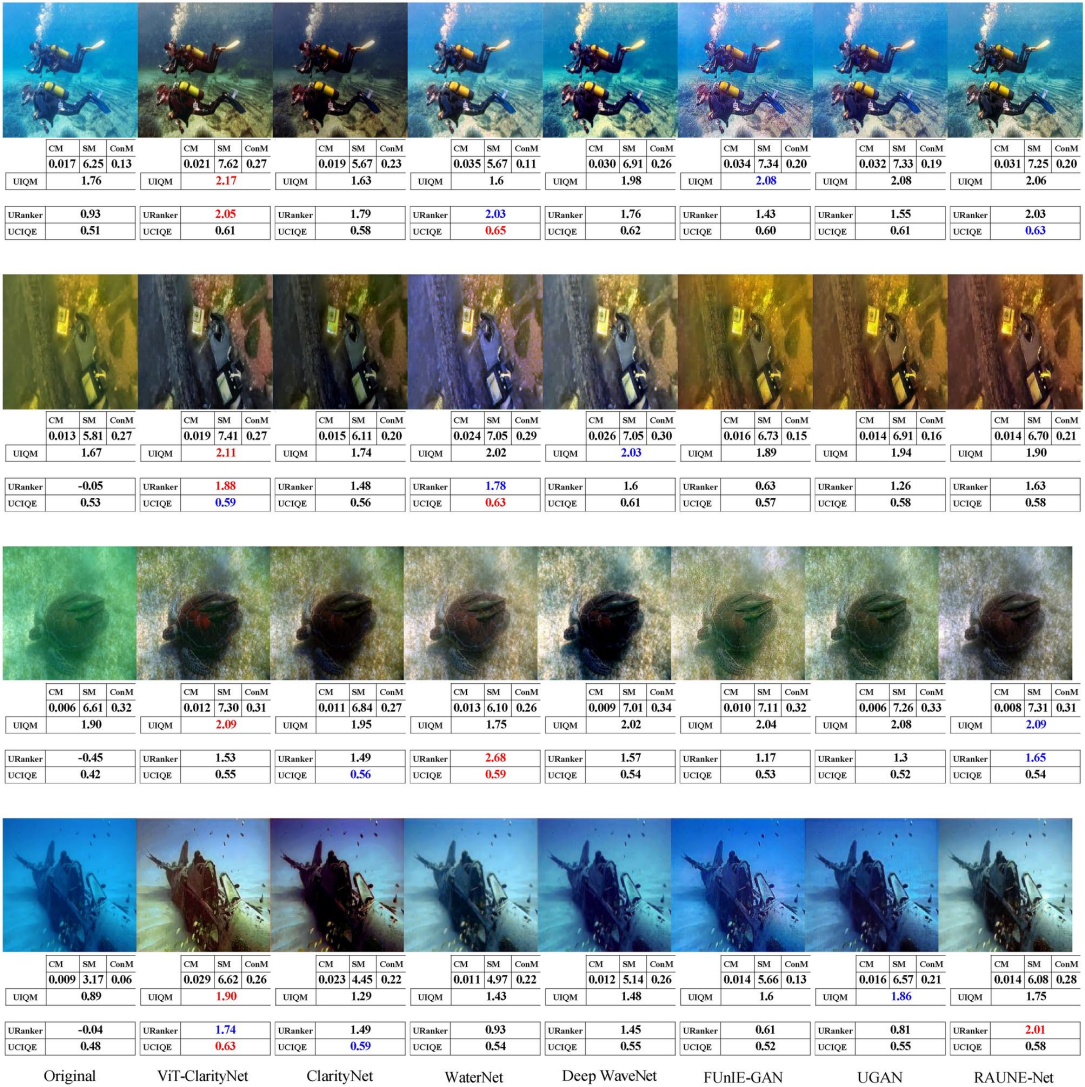
Visual comparison on samples from the EVUP dataset^33^. From left to right: raw underwater images, ViT-ClarityNet, ClarityNet, WaterNet^22^, Deep WaveNet^43^, FUnIE-GAN^33^, UGAN^32^ and RAUNE-Net^44^. Additionally, the CM, SM, ConM, UIQM, URanker, and UCIQUE metric scores are shown, with the best values highlighted in red and the second-best in blue.

**Fig. 10:**
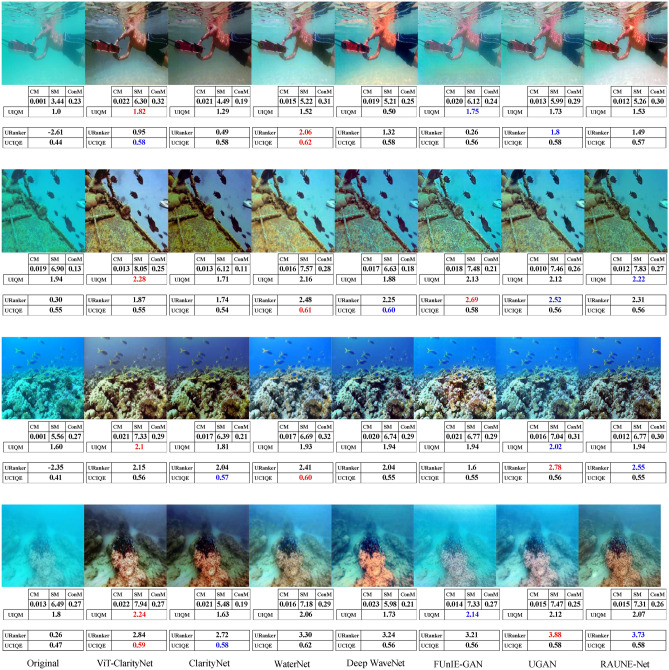
Visual comparison on samples from the SUIM dataset^45^. From left to right: raw underwater images, ViT-ClarityNet, ClarityNet, WaterNet^22^, Deep WaveNet^43^, FUnIE-GAN^33^, UGAN^32^ and RAUNE-Net^44^. Additionally, the CM, SM, ConM, UIQM, URanker, and UCIQUE metric scores are shown, with the best values highlighted in red and the second-best in blue.

**Fig. 11:**
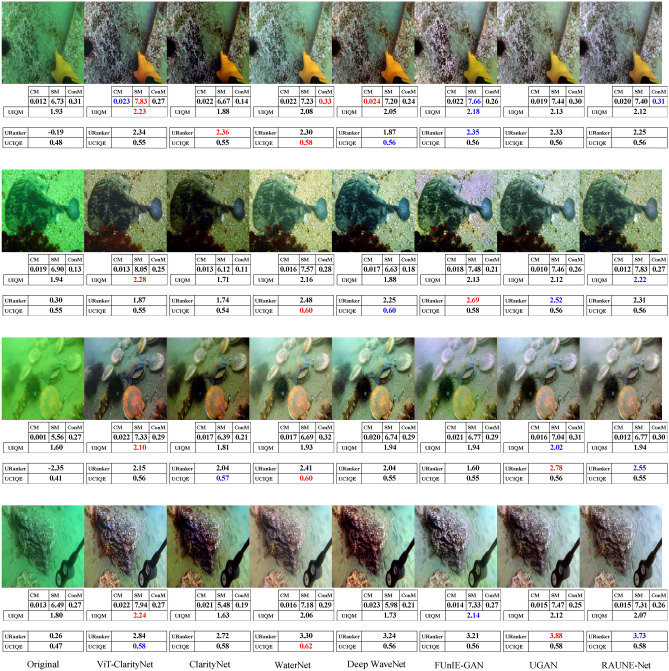
Visual comparison of samples from the U45 dataset^46^. From left to right: raw underwater images, ViT-ClarityNet, ClarityNet, Deep WaveNet^43^, RAUNE-Net^44^, FUnIE-GAN^33^, WaterNet^22^, and UGAN^32^. Additionally, the CM, SM, ConM, UIQM, URanker, and UCIQUE metric scores are shown, with the best values highlighted in red and the second-best in blue.

**Fig. 12:**
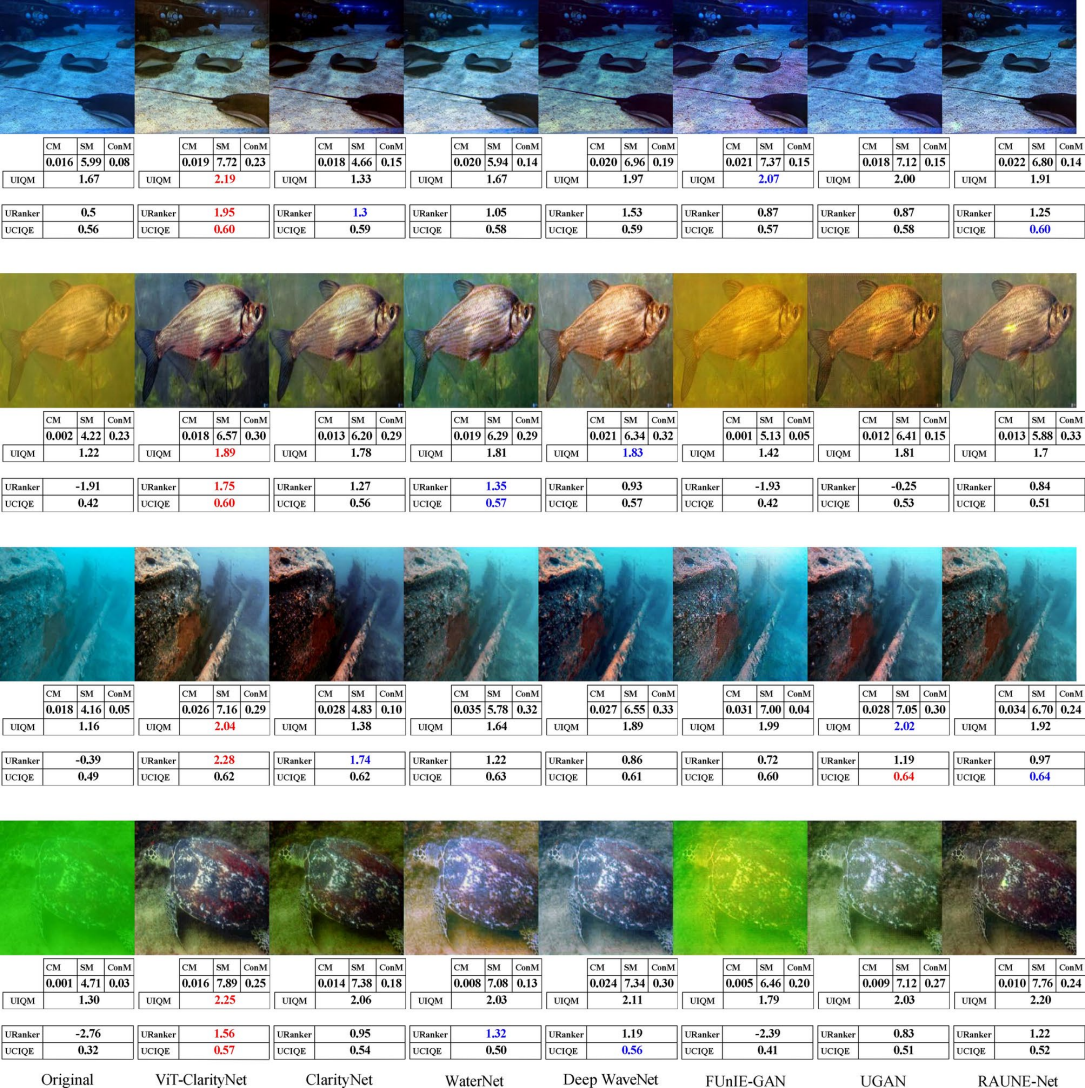
Visual comparison on samples from the UIEB dataset^22^. From left to right: raw underwater images, ViT-ClarityNet, ClarityNet, WaterNet^22^, Deep WaveNet^43^, FUnIE-GAN^33^, UGAN^32^ and RAUNE-Net^44^. Additionally, the CM, SM, ConM, UIQM, URanker, and UCIQUE metric scores are shown, with the best values highlighted in red and the second-best in blue.

**Fig. 13:**
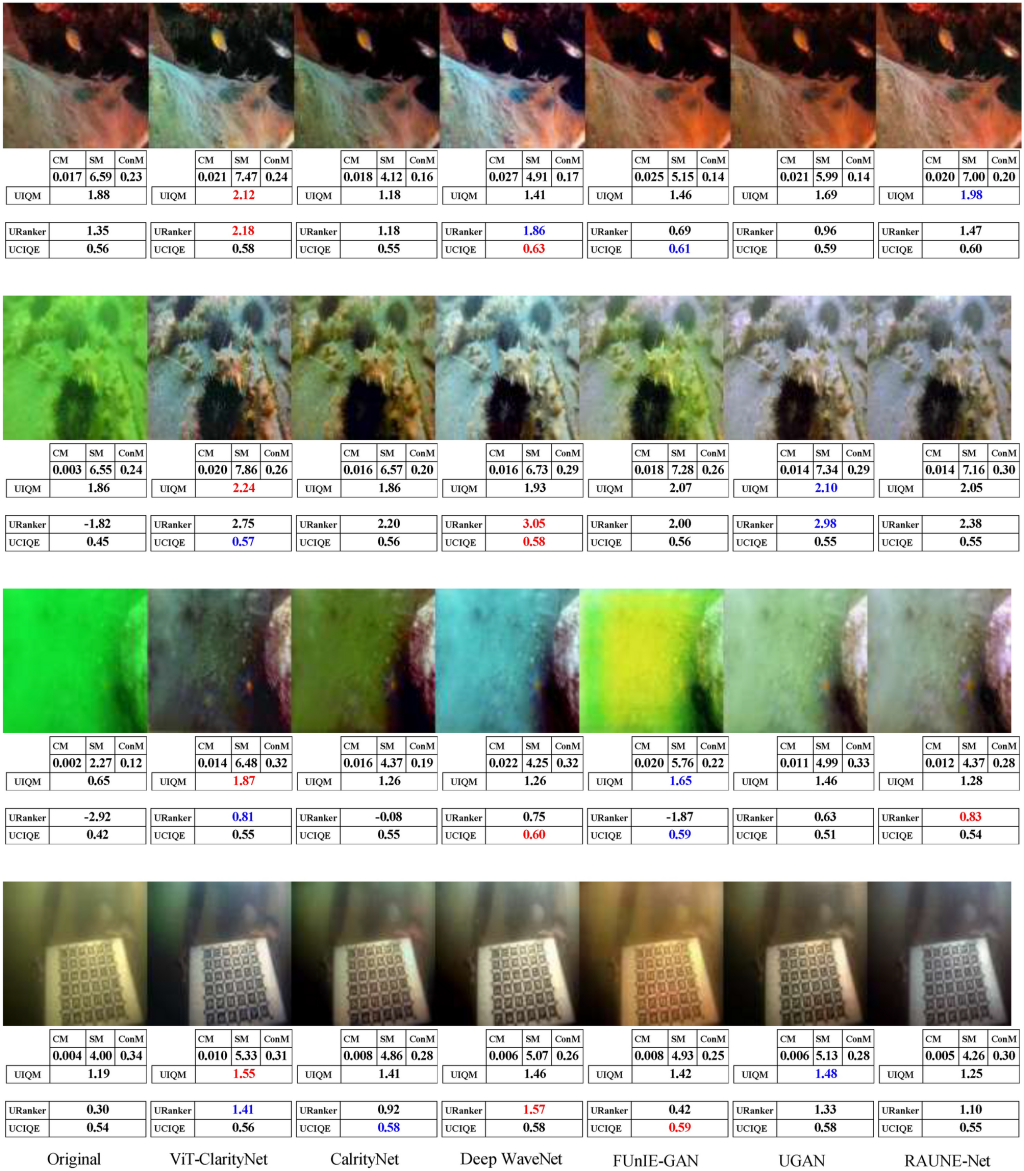
Visual comparison on samples from the USOD dataset^47^. From left to right: raw underwater images, ViT-ClarityNet, ClarityNet, Deep WaveNet^43^, FUnIE-GAN^33^, UGAN^32^, and RAUNE-Net^44^. Additionally, the CM, SM, ConM, UIQM, URanker, and UCIQUE metric scores are shown, with the best values highlighted in red and the second-best in blue.

To evaluate robustness against different illumination conditions, perceptual qualities, and underwater haze styles (bluish, greenish, and yellowish), samples from seven different datasets were utilized in this study: EVUP^33^ (Fig. 9), SUIM^45^ (Fig. 10), U45^46^ (Fig. 11), UIEB^22^ (Fig. 12), and USOD^47^ (Fig. 13). Quantitative evaluation against recent state-of-the-art methods, using the UCIQE metric^27^ on the UIEB^22^ and LSUI^48^ datasets is shown in Table 1. Additionally, Fig. 8 presents a visual evaluation of brightness variance on a sample from the Aquarium dataset^42^, comparing the ability to produce enhanced images with uniform brightness across all regions, avoiding uneven illumination.

**Table 1:** A qualitative comparison based on the UCIQE score^27^ was conducted against state-of-the-art methods, including Reti-Diff^49^, NU2Net^29^, ADP^50^, PUGAN^51^, and U-shape^48^.

Method	UIEB^22^	LSUI^48^	Average
Reti-Diff^49^	*0.628*	**0.633**	*0.631*
ADP^50^	0.611	0.619	0.615
PUGAN^51^	0.597	0.622	0.610
U-shape^48^	0.583	0.599	0.591
NU2Net^29^	0.576	0.608	0.592
ViT-ClarityNet	**0.638**	*0.627*	**0.633**
ClarityNet	0.588	0.612	0.600

The evaluation was performed on the UIEB^22^ and LSUI^48^ datasets, with the highest scores formatted in bold and the second-highest formatted in italic.

Additionally, the performance of the proposed image enhancement modules, ViT-ClarityNet and ClarityNet, is evaluated visually and quantitatively against recent state-of-the-art methods: Reti-Diff^49^, NU2Net^29^, ADP^50^, PUGAN^51^, U-shape^48^, Deep WaveNet^43^, RAUNE-Net^44^, FUnIE-GAN^33^, WaterNet^22^, and UGAN^32^.

For quantitative evaluation, three non-reference evaluation metrics are considered. The first two, UIQM^52^ and UCIQE^27^, are traditional metrics that are commonly used for evaluating underwater image enhancement. The UIQM score comprises three underwater image attribute measures: underwater image colorfulness (UICM), sharpness (UISM), and contrast (UIConM), where each attribute’s value is presented in the results to evaluate the sample’s quality based on these individual criteria. The UCIQE score provides an indication of the balance among chroma, saturation, and contrast in the output.

The final quantitative evaluation metric, URanker^29^, is a deep learning-based method used to assess image quality. This method employs a convolution-transformer architecture that evaluates images at multiple scales to capture both fine details and broader contextual information. The final quality score is obtained through a weighted summation of the outputs obtained from these different scales.

## Discussion

The feature enhancement capabilities are shown in Figs. 6 and 7. In Fig. 6, the enhanced images show a larger number of extracted SIFT keypoints, indicating clearer features. In Fig. 7, the object detection model successfully detected the shark only in the enhanced images from ViT-ClarityNet and ClarityNet, while failing to detect any objects in the original image. Despite being trained on the Aquarium dataset with underwater style, the model shows improved performance with enhanced images (without underwater style). This suggests that computer vision applications can achieve significant improvements when using enhanced images as input, even if the modules were trained on underwater datasets. Furthermore, this approach opens the possibility of using modules trained on in-air datasets for underwater applications.

To evaluate the effectiveness of vision transformers in generating global contextual features with a high receptive field in ViT-ClarityNet, it was observed that this approach led to more correlated pixel enhancements. As a result, the output of ViT- ClarityNet tends to minimize illumination variance across the image, ensuring a more uniform brightness and preventing regions from appearing overly bright or too dark. This effect is shown in Fig. 8 using a sample from the Aquarium dataset^42^, where the output of ViT-ClarityNet shows minimal brightness variance across the entire scene, which is additionally validated by compar- ing two selected regions in the image. Additionally, ViT-ClarityNet and ClarityNet effectively reduce blue casts, with ClarityNet being darker. Deep WaveNet^43^ offers balanced restoration with good contrast. FUnIE-GAN^33^ and UGAN^32^ enhance colors well but add a reddish hue. RAUNE-Net^44^ provides a well-balanced and slightly brighter restoration similar to Deep WaveNet^43^.

The comparisons shown in Figs. 9, 10, 11, 12, and 13 are analyzed based on several attributes: attenuation’s hue removal, colorfulness, and realism.

In terms of removing attenuation’s hue, although ViT-ClarityNet was trained to remove light blue attenuation only (Fig. 2), ViT-ClarityNet adapted to remove the various types of attenuation hues (dark blue, yellowish, greenish, and reddish). Deep WaveNet^43^ failed in the first row in Fig. 12. RAUNE-Net^44^, UGAN^32^, and WaterNet^22^ also performed well in this aspect; however, if the input image has a significant underwater attenuation, they may introduce a white fog effect, while in the same scenario, ClarityNet and FUnIE-GAN^33^ may introduce artifacts. The CM and SM values decreased, indicating that this effect decreased sharpness and colorfulness (e.g., Fig. 11 (third row) and Fig. 13 (third row)). Additionally, Deep WaveNet^43^, RAUNE-Net^44^, UGAN^32^, and WaterNet^22^ and FUnIE-GAN^33^ tended to remove attenuation only from the foreground, neglecting the background (e.g., Fig. 10 (fourth row) and Fig. 12 (third row)). ClarityNet showed good performance in removing the underwater’s hue, but it failed to enhance the image’s brightness (e.g., Fig. 9 (fourth row) and Fig. 11 (second row)).

In terms of colorfulness, if the input image has a significant underwater attenuation, ViT-ClarityNet and ClarityNet tend to result in more colorful images (e.g., Fig.13 (second row) and Fig. 11 (first and third rows)). However, they don’t produce as much color if the input image has moderate attenuation. These methods often result in less colorful outputs because they are trained to only remove underwater hues, which can also reduce overall colorfulness. Accordingly, the other methods generally produce more colorful images by maintaining some natural underwater colors (e.g., Fig. 9 (first row), Fig. 12 (third row), and Fig. 10 (third and fourth rows)).

In terms of realism, overly colorful images may be visually appealing but tend to look less realistic. For instance, in Fig. 10 (first row), Deep WaveNet^43^ and RAUNE-Net^44^ introduced a reddish hue to the swimmer’s skin. Additionally, RAUNE-Net^44^ produced images with uneven brightness, causing some areas to appear much shinier than others.

It is observed that UCIQE^27^ tends to give higher scores to images with greater brightness, neglecting artifacts in the results (e.g., Fig. 13 (fifth column, third row)). Additionally, Uranker^29^ was trained to maximize the quality score of reference images with a specific unattenuation style, which may not be optimal in all scenarios (e.g., Fig. 11 ViT-ClarityNet produces the most visually appealing result but does not achieve the highest Uranker score). Accordingly, visual evaluation results do not always align with non-reference quantitative metrics, and these metrics often disagree on the best performer. This variation explains the gap between the quantitative objective ratings and the human perception of visual quality.

## Conclusion

This paper focuses on underwater image enhancement, presenting ClarityNet and ViT-ClarityNet as solutions to improve image quality. ClarityNet is a straightforward encoder-decoder CNN, while ViT-ClarityNet integrates a vision transformer for more effective feature extraction. Evaluation of ViT-ClarityNet reveals its ability to produce images with consistent visual styles, benefiting from the transformer’s large receptive field. These image enhancement modules were tested against state-of-the-art methods using five real underwater datasets, evaluated through visual inspection and quality metrics such as UCIQE^27^, UIQM^52^, and URanker^29^. Additionally, the paper examines how image enhancement impacts object detection and SIFT matching performance.

To train these modules, the paper introduces BlueStyleGAN, a synthetic data generator that transfers attenuation effects from underwater images to in-air images. BlueStyleGAN is compared to state-of-the-art methods, showing superior training stability and more realistic style transfer through efficient use of depth maps. The generated image pairs (in-air images and their underwater counterparts) are then used to train the enhancement modules.

## Data Availability

The datasets generated and/or analyzed during this study are available from the corresponding author upon reasonable request. The images sourced from external datasets, including USOD [45], UIEB [22], EUVP [29], U45 [44], SUIM [43], SQUID [35], Aquarium[37], and KITTI [34], are publicly accessible through their respective websites. Furthermore, the enhanced outputs obtained for Vit-ClarityNet’s evaluation, as well as the synthetic underwater image datasets generated by BlueStyleGAN for training purposes, are publicly available at https://github.com/ME-1997/Vit-ClarityNet or can be requested from the corresponding author.
